# Multicenter validation of an explainable machine-learning model for APL, AMoL, and ALL

**DOI:** 10.3389/fonc.2026.1939329

**Published:** 2026-09-29

**Authors:** Xuanxuan Wang, Lixiang Ma, Zhongxin Wang, Wei Huang, Mengyu Zhang, Yuanyuan Hu

**Affiliations:** 1 Department of Clinical Laboratory, The First Affiliated Hospital of Anhui Medical University, Hefei, Anhui, China; 2 Department of Clinical Laboratory, The Affiliated Guangdong Second Provincial General Hospital of Jinan University, Guangzhou, Guangdong, China; 3 Department of Clinical Laboratory, Tongling People’s Hospital, Tongling, Anhui, China; 4 Department of Clinical Laboratory, Wanbei Coal Electric Group General Hospital, Suzhou, Anhui, China

**Keywords:** acute leukemia, clinical decision support, machine learning, random forest, routine hematological parameters, SHAP

## Abstract

**Background:**

Acute leukemias require rapid subtype identification to guide timely treatment. Differentiation of acute lymphoblastic leukemia (ALL), acute monocytic leukemia (AMoL/M5), and acute promyelocytic leukemia (APL/M3) often relies on specialized tests that may not be immediately available. This study evaluated differentiation only among patients already confirmed to have one of these three diagnoses, rather than screening an unselected population with suspected acute leukemia.

**Methods:**

This multicenter retrospective study included 9,398 newly diagnosed, previously untreated patients with established APL/M3, AMoL/M5, or ALL between May 2019 and May 2025 at four tertiary hospitals in China. Relapse, remission, follow-up, and non-initial disease episodes were excluded. Twenty-four routine hematological parameters were evaluated. One center served as the development cohort with five-fold stratified cross-validation, while three independent centers were reserved for external validation. Ten machine-learning algorithms were compared. Performance was assessed using AUC, sensitivity, specificity, accuracy, positive predictive value (PPV), negative predictive value (NPV), and F1 score. SHapley Additive exPlanations (SHAP) supported feature selection and interpretation.

**Results:**

An 18-feature random forest model achieved a development-stage cross-validated AUC of 0.93 and external AUCs of 0.89, 0.87, and 0.90. Overall sensitivity, specificity, PPV, NPV, accuracy, and F1 score were 0.78, 0.90, 0.81, 0.91, 0.81, and 0.79, respectively. SHAP identified leukocyte, erythrocyte, and platelet indices as the most important features. A Streamlit application was developed for clinical evaluation. Using a development-derived APL alert threshold of 0.0966, APL sensitivity was 99.02% in development and 96.32%–97.94% across external cohorts, with PPVs of 54.34% and 35.58%–84.25%, respectively. External validation showed center-specific calibration shifts, indicating that predicted probabilities should not be interpreted as universally calibrated absolute diagnostic probabilities.

**Conclusions:**

Among patients already confirmed to have APL/M3, AMoL/M5, or ALL, this explainable model enabled low-cost preliminary differentiation using routine hematological data. It may support subtype prioritization and confirmatory testing but cannot replace standard diagnostics, identify other diagnoses, or screen unselected patients. Prospective multinational and workflow-based validation is required before routine implementation.

## Introduction

Acute leukemia (AL) is a malignant clonal disorder of hematopoietic progenitor cells characterized by the uncontrolled proliferation of blasts and immature cells in the bone marrow. Their accumulation suppresses normal hematopoiesis and may be accompanied by infiltration of extramedullary organs, including the liver, spleen, and lymph nodes (1). According to the lineage of the malignant cells, AL is broadly classified as acute myeloid leukemia (AML) or acute lymphoblastic leukemia (ALL) (2). Globally, AML causes more than 80,000 deaths annually, and this burden is projected to increase substantially over the next two decades (3). In the United States, an estimated 6,250 new cases of ALL and 1,600 related deaths are projected for 2026 (4). Delayed recognition and diagnosis may contribute to poor outcomes, particularly in settings with limited specialist resources (5).

AML comprises biologically and clinically distinct subtypes that influence prognosis and treatment selection (6). Acute promyelocytic leukemia (APL/M3) is a distinctive AML subtype that occurs frequently in adolescents and young adults and accounts for approximately 5%–20% of AML cases (7). It is characterized by severe coagulopathy, a high risk of hemorrhage, and a substantial risk of early death (8). Although contemporary treatment has made APL one of the most curable forms of acute leukemia, early mortality remains an important clinical problem (9). Early death, commonly defined as death within 30 days of presentation, occurs in approximately 5%–29% of patients and is often caused by thrombohemorrhagic complications, including intracranial hemorrhage related to coagulopathy at diagnosis (9–11).

APL is therefore a hematologic emergency in which rapid recognition and immediate initiation of appropriate supportive and disease-specific therapy are essential (12). Acute monocytic leukemia (AMoL/M5), another AML subtype, is also associated with an unfavorable prognosis, with reported 3-year disease-free and overall survival rates of approximately 26% and 31%, respectively (13). AMoL/M5 is frequently associated with leukocytosis, extramedullary infiltration, leukostasis, and disseminated intravascular coagulation, all of which may require urgent intervention (14). Outcomes in ALL vary markedly by age: although 5-year overall survival approaches 90% in pediatric patients, it remains substantially lower in older adults (15). Corticosteroids may be used to reduce lymphoblast proliferation before definitive chemotherapy in selected patients (16).

The diagnosis of AL relies on integrated cytomorphological, immunophenotypic, cytogenetic, and molecular assessment. In centers with experienced staff and continuous microscopy services, peripheral-blood or bone-marrow morphology can rapidly identify characteristic APL features and remains an essential component of the diagnostic workflow (17–19). However, immediate specialist morphological review may not be available during off-hours or in resource-limited institutions, and completion of the confirmatory diagnostic workup may still require additional time. Within a population already confirmed to have APL/M3, AMoL/M5, or ALL, an automated signal derived from available complete blood count data could help prioritize subtype-specific smear review and confirmatory testing, but should not replace microscopy.

Alcazer et al. recently developed the Artificial Intelligence Prediction of Acute Leukemia (AI-PAL) tool to distinguish APL, non-APL AML, and ALL using age and routine hematological, biochemical, and coagulation parameters in a multicenter French cohort (20). This study established the feasibility of laboratory-data-based acute leukemia classification. However, whether parameters derived exclusively from routine complete blood counts can distinguish AMoL/M5, a specific AML subtype, from APL/M3 and ALL in a large East Asian multicenter population remains insufficiently studied.

We hypothesized that routinely available clinical laboratory parameters contain sufficient information to support automated preliminary differentiation among APL/M3, AMoL/M5, and ALL in patients already confirmed to have one of these three diagnoses. We therefore systematically compared multiple machine-learning algorithms using routine hematological data, applied SHAP-based feature selection to improve parsimony and interpretability, and developed a random forest model as an adjunct for prioritizing subsequent morphological and confirmatory evaluation within this restricted diagnostic population. The model was not designed or validated as a general screening tool for unselected patients with suspected acute leukemia.

## Methods

### Study population

This multicenter retrospective study aimed to develop and validate an interpretable model for differentiating APL/M3, AMoL/M5, and ALL among patients already confirmed to have one of these three diagnoses. It was not designed to distinguish these categories from other AML subtypes, mixed-phenotype acute leukemia, other hematological malignancies, or non-malignant conditions. We enrolled eligible patients diagnosed between May 1, 2019, and May 31, 2025, at four tertiary hospitals in China: The First Affiliated Hospital of Anhui Medical University, Wanbei Coal Electric Group General Hospital, Tongling People’s Hospital, and Guangdong Second Provincial General Hospital. The protocol, including retrospective use of data from all four participating centers, was reviewed and approved under the coordinating ethics approval of the Clinical Research Ethics Committee of The First Affiliated Hospital of Anhui Medical University (approval No. PJ2025-02-19). The requirement for individual informed consent was waived by the committee because the study used de-identified, routinely collected retrospective data. The study was conducted in accordance with the Declaration of Helsinki.

The reference diagnosis was established at each participating institution by experienced hematologists and hematopathologists according to the 2016 World Health Organization classification, using integrated review of peripheral-blood and/or bone-marrow morphology, flow-cytometric immunophenotyping, cytogenetic findings, and molecular results. APL was confirmed by demonstration of PML::RARA or the corresponding t(15;17) rearrangement. AMoL/M5 and ALL were assigned on the basis of concordant morphological and lineage-specific immunophenotypic findings, supported by cytogenetic or molecular testing when indicated. The same diagnostic framework and eligibility requirements were applied across all four institutions, and cases without sufficient documentation for definitive subtype assignment were excluded.

### Data collection and processing

Demographic characteristics and routine hematological results were extracted from the electronic medical record systems. Eligibility was restricted to patients with a first confirmed diagnosis of APL/M3, AMoL/M5, or ALL according to the 2016 World Health Organization classification of acute leukemia (21). *De novo* status was established by review of the documented medical history and diagnostic episode, and only the index complete blood count associated with the initial diagnosis was eligible. Records from patients with relapsed or previously treated disease, complete or partial remission, follow-up or subsequent disease episodes, or insufficient history to verify a first diagnosis were excluded before construction of the analytic dataset. Thus, no relapse-phase or remission-phase records were used for model development, fold-wise validation, or external validation. Missingness was quantified separately for each candidate variable in the development cohort. All 24 prespecified candidate variables were retained; no variable was excluded solely on the basis of missingness. All variable-level missing-data rates were below 20% (range, 0.01%–16.74%) and are reported in **Supplementary Table S8**. Missing values were imputed using the mean. Within each cross-validation iteration, imputation values were estimated exclusively from the four training folds and subsequently applied to the held-out validation fold. After model selection, imputation values were re-estimated using the complete development cohort and applied unchanged to the external validation datasets. The final candidate set comprised 24 routine parameters: white blood cell count (WBC; 10^9^/L); absolute and percentage counts of neutrophils, eosinophils, basophils, lymphocytes, and monocytes; red blood cell count (RBC; 10¹²/L); red cell distribution width coefficient of variation (RDW-CV; %); red cell distribution width standard deviation (RDW-SD; fL); hemoglobin (g/L); hematocrit (HCT; %); mean corpuscular hemoglobin (MCH; pg); mean corpuscular volume (MCV; fL); mean corpuscular hemoglobin concentration (MCHC; g/L); platelet count (PLT; 10^9^/L); plateletcrit (PCT; %); platelet distribution width (PDW; fL); mean platelet volume (MPV; fL); and platelet large-cell ratio (P-LCR; %).

Unique participants were identified within each institution using the hospital-specific medical-record identifier before de-identification. Records were grouped by patient identifier and diagnostic episode. When more than one complete blood count was available during the index diagnostic episode, only the earliest pretreatment result temporally closest to the initial diagnostic work-up was retained; subsequent or repeated measurements from the same episode were discarded. Each patient therefore contributed only one observation to the analytic dataset. Patient identifiers were then replaced with study codes before analysis.

The predictor set was intentionally restricted to parameters derived from the complete blood count. This restriction was chosen to evaluate the discriminatory information available from a single, routinely generated laboratory test and to ensure consistent predictor availability across all four participating centers. Although lactate dehydrogenase and coagulation parameters such as prothrombin time and fibrinogen are clinically relevant in the initial evaluation of suspected acute leukemia, particularly APL, these variables were not consistently available at the same index time point in the harmonized retrospective datasets and were therefore not included in the present model.

Data from The First Affiliated Hospital of Anhui Medical University constituted the development cohort (n = 7,077) and were analyzed using five-fold stratified cross-validation. No separate fixed internal test cohort was created. In each iteration, four folds (80%) were used for model fitting and the remaining fold (20%) was used for fold-wise validation. Independent cohorts from Wanbei Coal Electric Group General Hospital, Tongling People’s Hospital, and Guangdong Second Provincial General Hospital were reserved exclusively for external validation and remained unavailable during all model-development and feature-selection procedures. Hematological parameters were measured using Sysmex XN-9000 automated hematology analyzers.

### Model development and comparison

The 24 candidate hematological parameters were used to train and compare ten machine-learning algorithms: random forest (RF), extreme gradient boosting (XGBoost), adaptive boosting (AdaBoost), gradient boosting (GB), light gradient boosting machine (LightGBM), logistic regression (LR), multilayer perceptron (MLP), decision tree (DT), support vector machine (SVM), and k-nearest neighbors (KNN).

Hyperparameters were optimized using a prespecified grid search with five-fold stratified cross-validation in the development cohort. Macro-averaged one-vs-rest AUC was used as the optimization metric. In each iteration, all preprocessing procedures, including mean imputation and, where applicable, z-score standardization, were fitted exclusively using the four training folds and then applied to the held-out validation fold. The same cross-validation fold assignments were used for all candidate algorithms. No separate fixed internal test cohort was used. The three external validation datasets were not used for preprocessing, feature ranking, backward feature elimination, hyperparameter optimization, algorithm comparison, or model selection. The prespecified search spaces are provided in **Supplementary Table S10**. Model performance was evaluated using AUC, sensitivity, specificity, positive predictive value (PPV), negative predictive value (NPV), accuracy, and F1 score.

To assess whether differences in variable scales affected the comparison among algorithms, we performed an additional standardization sensitivity analysis. Each of the ten algorithms was evaluated with and without z-score standardization using five-fold stratified cross-validation, with shuffling and a fixed random seed of 42. The same cross-validation partitions and model hyperparameters were used under both preprocessing conditions. Within each fold, the StandardScaler was fitted exclusively to the training partition and subsequently applied to the corresponding validation partition to prevent information leakage. The influence of standardization was quantified as the difference in macro-averaged one-vs-rest AUC between the standardized and unscaled analyses.

The robustness of the final model to the choice of imputation method was evaluated by comparing mean, median, and random-forest-based imputation using five-fold stratified cross-validation. Within each fold, all imputation procedures were fitted exclusively to the training partition. Performance was summarized using the macro-averaged one-vs-rest AUC. The complete results of this sensitivity analysis are provided in **Supplementary Table S9**.

Paired DeLong tests based on the development-stage cross-validated predictions were used to compare AUCs among the ten candidate models, with P < 0.05 indicating statistical significance. RF, XGBoost, and LightGBM were retained for further feature-reduction assessment because they had the three highest observed cross-validated AUCs and their pairwise AUC differences were small. A nonsignificant DeLong result was interpreted only as absence of a statistically detectable difference and not as evidence of equivalence or noninferiority. After all development decisions had been completed, the locked final model was fitted using the complete development cohort and evaluated without modification in the three independent external cohorts.

### Feature selection and model interpretation

Routine hematological datasets contain correlated and potentially redundant variables. Feature reduction can decrease noise, simplify the model, and improve interpretability. SHapley Additive exPlanations (SHAP) were used to quantify and visualize model-attributed feature importance. SHAP values describe associations between predictor values and the model output and were not interpreted as causal effects.

SHAP feature importance was calculated independently within each cross-validation training partition. In each iteration, the fold-specific model and all preprocessing steps were fitted using only the four training folds, and the mean absolute SHAP value of each feature was calculated from that training partition. For each feature, the fivefold-specific mean absolute SHAP values were then averaged to produce a consensus SHAP importance score and a unified feature ranking. Backward elimination followed this consensus ranking by iteratively removing the least important variable, generating models containing between 24 and 1 feature. No external-validation data were used to calculate SHAP values or determine the ranking.

Each SHAP-ranked reduced model was evaluated using five-fold stratified cross-validation in the development cohort. DeLong’s test was used to compare the paired cross-validated predictions from each reduced model with those from the corresponding full 24-feature model. A two-sided P value < 0.05 was considered statistically significant. P values were interpreted as tests of statistical difference only and were not used to claim equivalence or noninferiority. The primary model was selected by considering parsimony together with the observed AUC trajectory across the backward-elimination path. Because this procedure does not specifically remove clinically related predictors, a separate collinearity assessment was subsequently performed.

### Assessment of predictor collinearity

Potential collinearity among the 18 retained predictors was assessed in the development cohort using Spearman correlation coefficients and variance inflation factors (VIFs) after mean imputation. A VIF greater than 5 was considered indicative of potentially important collinearity. Because random forests use recursive partitioning, bootstrap aggregation, and random predictor subsampling rather than regression-coefficient estimation, correlated predictors do not produce the coefficient instability associated with conventional regression models. Nevertheless, correlated variables can substitute for one another during tree splitting and share feature-importance or SHAP attribution; therefore, individual importance values were interpreted cautiously. The number of features in the final model was determined by considering parsimony together with the observed development-stage cross-validated AUC trajectory along the original SHAP-ranked backward-elimination path. The nonsignificant DeLong result for the 18-feature model was not interpreted as evidence of equivalence or noninferiority.

### APL-prioritized alert-threshold analysis

Because delayed recognition of APL/M3 has more urgent clinical consequences than misclassification between the other modeled subtypes, an APL-prioritized alert analysis was performed using the probability assigned to APL by the unchanged final random forest model. For this reviewer-requested clinically oriented analysis, the high-sensitivity target was defined as at least 99% sensitivity for APL in the development-center cohort. The highest probability threshold satisfying this target was selected, thereby maximizing specificity among thresholds meeting the sensitivity criterion. This threshold was then locked and applied unchanged to each external validation cohort; no external cohort was used to select or modify the threshold. APL was treated as the positive class and AMoL/M5 and ALL as the negative class. Sensitivity, specificity, positive predictive value (PPV), negative predictive value (NPV), and the number of missed APL cases were calculated, with 95% confidence intervals estimated using the Wilson method.

### Calibration assessment

Calibration was assessed for the final 18-feature random forest model using one-vs-rest analyses for APL/M3, AMoL/M5, and ALL. In the development cohort, calibration was evaluated from the pooled out-of-fold probabilities generated by five-fold stratified cross-validation. In each external-validation cohort, probabilities were obtained from the locked model fitted to the complete development cohort; no recalibration or center-specific updating was performed. Calibration plots compared observed proportions with mean predicted probabilities across 10 quantile-based groups. Calibration-in-the-large (intercept; ideal value, 0) and calibration slope (ideal value, 1) were estimated by logistic recalibration. Class-specific Brier scores and an overall multiclass Brier score, defined as the mean sum of squared differences between the three predicted probabilities and one-hot outcomes, were calculated, with lower values indicating better probabilistic accuracy. Wald 95% confidence intervals were used for calibration intercepts and slopes, and 95% confidence intervals for Brier scores were estimated using 2,000 class-stratified bootstrap replicates with a fixed random seed of 42.

### Development of the Streamlit web application

To facilitate clinical evaluation and future translation within the restricted three-category population, a web-based application was developed in PyCharm 2023.3.5 and deployed using Streamlit 1.34.0. After the 18 required laboratory parameters are entered, the application calculates the predicted probabilities of APL/M3, AMoL/M5, and ALL and displays the subtype with the highest predicted probability. The application is not intended for general screening of unselected patients with suspected acute leukemia because every input is necessarily assigned to one of the three modeled categories. The application independently compares the APL probability with the prespecified threshold of 0.0966, regardless of which subtype has the highest probability. When the APL probability is at or above 0.0966, it displays an “APL ALERT—APL cannot be excluded” message and prompts urgent peripheral-blood smear review, coagulation assessment, PML::RARA testing, and specialist hematology assessment. A permanent safety notice states that an APL probability below 0.0966 must not be used to rule out APL and that the output cannot replace morphology, flow cytometry, cytogenetics, molecular confirmation, or specialist interpretation.

### Statistical analysis

Statistical analyses were performed using SPSS version 26.0 (IBM Corp.) and Python 3.11. Categorical variables are presented as counts and percentages and were compared using the chi-square test. Continuous variables are presented as the mean ± standard deviation when normally distributed and as the median with interquartile range when non-normally distributed. Comparisons among the three leukemia subtypes were performed using one-way analysis of variance or the Kruskal–Wallis test, as appropriate. DeLong’s test was used to compare AUCs. All tests were two-sided, and P < 0.05 was considered statistically significant.

In each external validation cohort, performance was additionally calculated separately for APL/M3, AMoL/M5, and ALL using a one-vs-rest definition. Class-specific AUC, sensitivity, specificity, PPV, NPV, F1 score, and the number of reference-standard cases were reported. Ninety-five percent confidence intervals for class-specific AUCs were estimated using 2,000 class-stratified bootstrap replicates with a fixed random seed of 42, and Wilson confidence intervals were calculated for sensitivity and PPV.

## Results

### Patient characteristics

After screening the larger source-record pool and excluding relapse, remission, previously treated, follow-up, non-initial, and unverifiable disease episodes, 9,398 newly diagnosed patients were included between May 1, 2019, and May 31, 2025. No relapsed or remission-phase cases were present in the analytic cohort. The development-center cohort comprised 7,077 patients from The First Affiliated Hospital of Anhui Medical University, including 2,043 with APL/M3, 1,783 with AMoL/M5, and 3,251 with ALL; this cohort was used for five-fold stratified cross-validation, with four folds used for model fitting and one fold used for validation in each iteration. No separate fixed internal test cohort was created. The external validation cohort included 2,321 patients from three institutions: 434 from Wanbei Coal Electric Group General Hospital (APL/M3, n = 97; AMoL/M5, n = 116; ALL, n = 221), 1,024 from Tongling People’s Hospital (APL/M3, n = 761; AMoL/M5, n = 32; ALL, n = 231), and 863 from Guangdong Second Provincial General Hospital (APL/M3, n = 428; AMoL/M5, n = 7; ALL, n = 428).

The study design is summarized in **Figure 1**. Baseline characteristics of the development-center cohort are shown in **Table 1**. The median ages were 46 years in the APL/M3 group, 44 years in the AMoL/M5 group, and 43 years in the ALL group; the proportions of male patients were 43.0%, 44.4%, and 46.1%, respectively. Characteristics of the external validation cohorts are provided in **Supplementary Tables S1**–**S3**.

**Figure 1:**
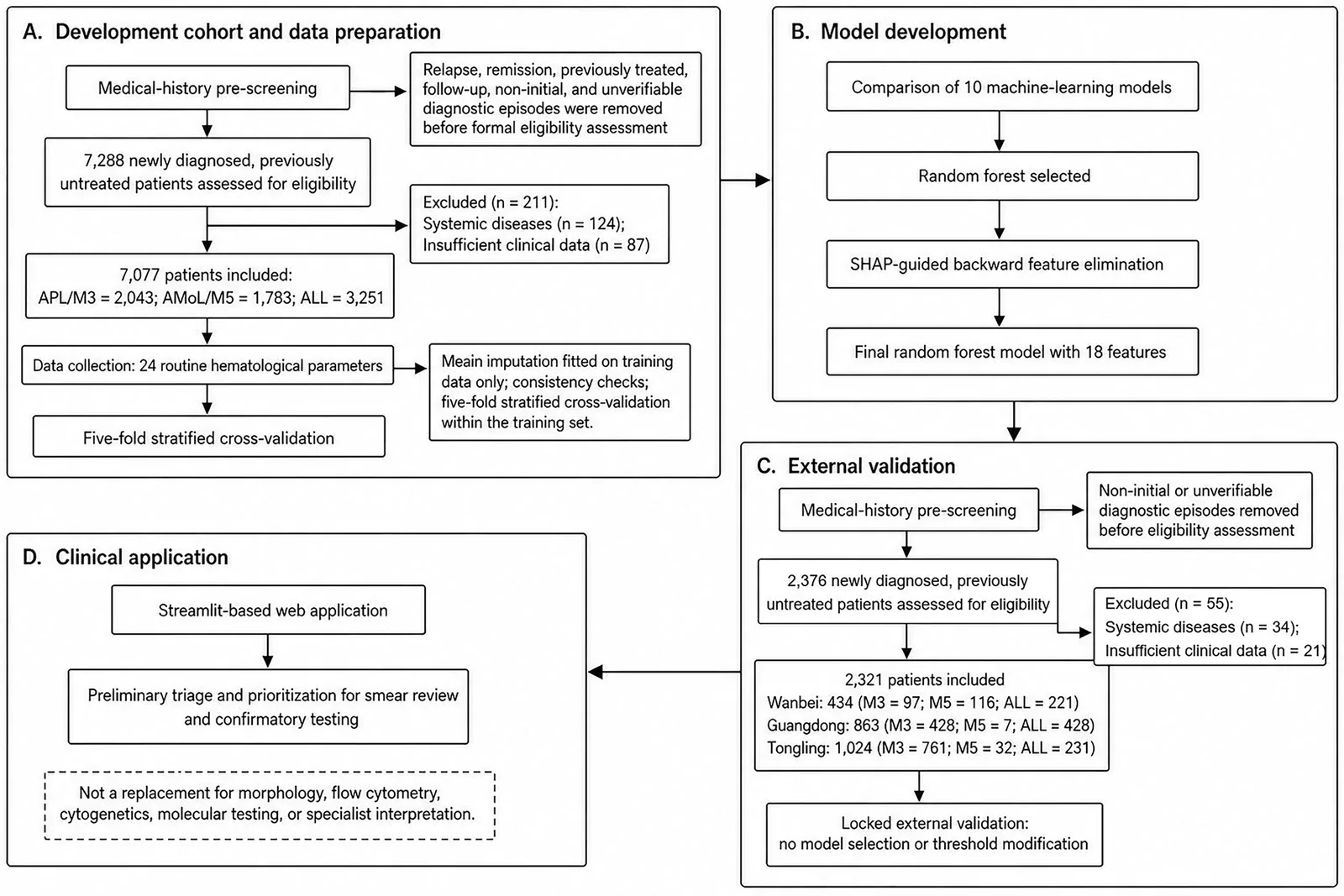
Flowchart of the study design. **(A)** Development-cohort selection and data preparation. **(B)** Comparison of machine-learning algorithms and SHAP-guided feature selection for the final 18-feature random forest model. **(C)** Independent external validation in the Wanbei, Guangdong, and Tongling cohorts. **(D)** Streamlit-based implementation for adjunctive subtype prioritization and confirmatory testing.

**Table 1:** Baseline characteristics of the development-center cohort.

Characteristic	Group			p-value
	M3 N = 2,043	M5 N = 1,783	ALL N = 3,251	
age, Median (Q1, Q3)	46 (31, 52)	44 (29, 55)	43 (26, 56)	0.113
sex, n (%)				0.084
Male	879 (43.0%)	792 (44.4%)	1,499 (46.1%)	
Female	1,164 (57.0%)	991 (55.6%)	1,752 (53.9%)	
BASO#	0.020 (0.010, 0.030)	0.010 (0.000, 0.020)	0.010 (0.000, 0.020)	<0.001
BASO(%)	0.30 (0.10, 0.60)	0.14 (0.00, 0.50)	0.20 (0.00, 0.50)	<0.001
EO#	0.06 (0.02, 0.13)	0.01 (0.00, 0.06)	0.01 (0.00, 0.03)	<0.001
EO(%)	1.20 (0.30, 2.90)	0.34 (0.00, 2.00)	0.10 (0.00, 1.04)	<0.001
HCT(%)	34 (23, 39)	26 (19, 33)	26 (21, 32)	<0.001
HGB, g/L	110 (76, 128)	87 (62, 107)	87 (71, 105)	<0.001
Lymphocyte#	1.28 (0.84, 1.84)	0.74 (0.41, 1.07)	0.63 (0.28, 1.05)	<0.001
Lymphocyte(%)	29 (20, 38)	29 (16, 56)	26 (11, 49)	<0.001
MCH, pg	31.10 (29.80, 32.40)	31.70 (30.30, 33.00)	29.70 (28.40, 32.70)	<0.001
MCHC, g/L	332 (324, 340)	335 (324, 347)	333 (322, 345)	<0.001
MCV, fL	93 (90, 97)	94 (90, 99)	87 (85, 102)	<0.001
MONO	0.30 (0.16, 0.48)	0.24 (0.03, 0.50)	0.21 (0.03, 0.48)	<0.001
MONO %	7 (3, 9)	7 (3, 14)	7 (2, 13)	<0.001
MPV, fL	10.90 (10.20, 11.80)	10.90 (10.30, 11.50)	10.90 (10.50, 11.40)	0.350
Neutrophil	2.8 (1.6, 4.6)	1.7 (0.3, 2.9)	1.7 (0.5, 3.4)	<0.001
Neutrophil(%)	59 (48, 70)	54 (20, 68)	60 (35, 77)	<0.001
P-LCR(%)	31 (26, 38)	31 (27, 36)	31 (29, 35)	0.080
PCT(%)	0.18 (0.12, 0.25)	0.14 (0.05, 0.14)	0.14 (0.09, 0.16)	<0.001
PDW, fL	12.20 (11.00, 14.40)	12.00 (10.80, 13.10)	12.00 (11.10, 13.00)	<0.001
PLT, 10^9^/L	159 (54, 227)	47 (14, 121)	71 (24, 151)	<0.001
RBC, 10^12^/L	3.60 (2.47, 4.26)	2.76 (1.99, 3.44)	2.86 (2.32, 3.45)	<0.001
RDW-CV(%)	14.60 (13.30, 16.20)	15.00 (13.80, 16.90)	15.10 (13.60, 17.00)	<0.001
RDW-SD, fL	49 (44, 55)	51 (46, 58)	48 (42, 60)	<0.001
WBC, 10^9^/L	5 (3, 7)	3 (1, 5)	3 (1, 6)	<0.001

Continuous variables are presented as median (interquartile range), and categorical variables are presented as number (percentage).

WBC, white blood cell count; RBC, red blood cell count; HGB, hemoglobin; HCT, hematocrit; MCV, mean corpuscular volume; MCH, mean corpuscular hemoglobin; MCHC, mean corpuscular hemoglobin concentration; RDW-SD, red cell distribution width standard deviation; RDW-CV, red cell distribution width coefficient of variation; PLT, platelet count; PCT, plateletcrit; PDW, platelet distribution width; MPV, mean platelet volume; P-LCR, platelet large-cell ratio.

### Model development and performance comparison

Ten machine-learning algorithms were evaluated using the 24 routine hematological parameters and five-fold stratified cross-validation in the development cohort to differentiate APL/M3, AMoL/M5, and ALL. As shown in **Figures 2A–J**, XGBoost (AUC = 0.9390), RF (AUC = 0.9378), and LightGBM (AUC = 0.9314) achieved the highest development-stage cross-validated discrimination. The remaining models achieved AUCs of 0.9231 for GB, 0.8456 for AdaBoost, 0.8454 for DT, 0.8240 for SVM, 0.8183 for KNN, 0.6995 for MLP, and 0.6847 for LR.

**Figure 2:**
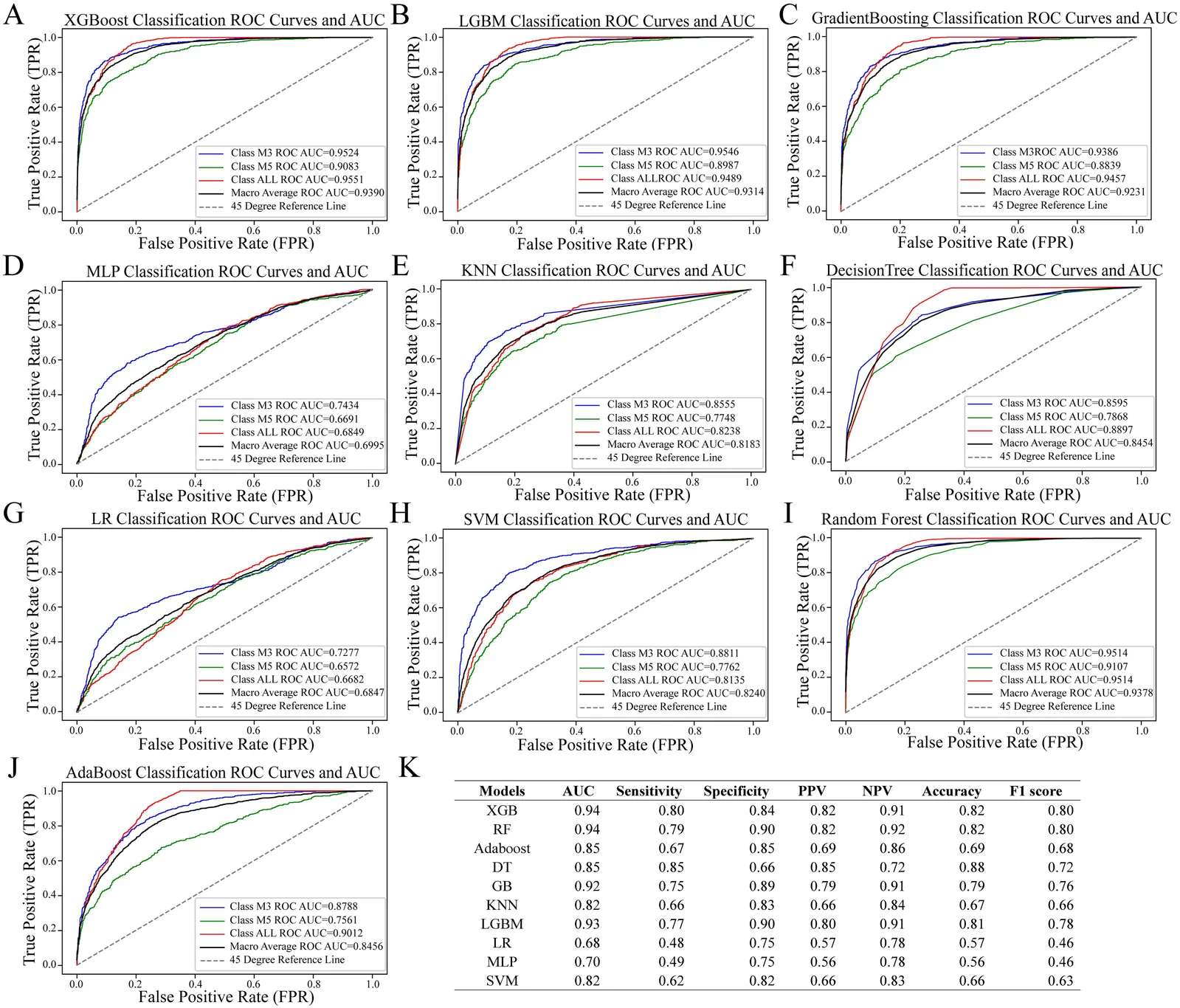
Five-fold cross-validated performance of the machine-learning models in the development cohort. **(A–J)** Receiver operating characteristic curves based on development-stage cross-validated predictions for the ten machine-learning models. **(K)** Comparison of the corresponding mean AUC, sensitivity, specificity, accuracy, positive predictive value (PPV), negative predictive value (NPV), and F1 score across models.

Precision–recall curves and confusion matrices based on the development-stage cross-validated predictions for all models are presented in **Supplementary Figures S1** and **S2**. **Figure 2K** summarizes the corresponding AUC, sensitivity, specificity, accuracy, PPV, NPV, and F1 score for each algorithm.

Pairwise DeLong tests based on the development-stage cross-validated predictions were used to compare model AUCs (**Supplementary Table S4**). No statistically significant differences were detected among RF, LightGBM, and XGBoost (all P > 0.05); these findings were not interpreted as evidence of equivalence or noninferiority. These three algorithms were retained for feature-reduction analysis because they had the highest observed cross-validated AUCs, whereas the other algorithms showed lower observed discrimination.

In the standardization sensitivity analysis, z-score standardization increased the macro-averaged AUC by 0.1091 for MLP, 0.0786 for SVM, and 0.0418 for KNN. In contrast, the AUC changes were small for LightGBM (+0.0011), RF (+0.0002), decision tree (+0.0002), gradient boosting (<+0.0001), AdaBoost (<+0.0001), and XGBoost (0.0000). A small decrease was observed for LR (−0.0029). Although standardization improved several scale-sensitive algorithms, XGBoost, LightGBM, and RF remained the three best-performing algorithms. Therefore, the models retained for subsequent feature-reduction analysis were unchanged (**Supplementary Table S7**).

The final RF model showed similar discrimination across the three imputation strategies. The five-fold macro-averaged AUCs were 0.9415 with mean imputation, 0.9414 with median imputation, and 0.9399 with random-forest-based imputation. Compared with mean imputation, the AUC differences were -0.0001 for median imputation and -0.0016 for random-forest-based imputation. Thus, random-forest-based imputation did not improve model performance, and mean imputation was retained (**Supplementary Table S9**).

### Feature reduction and selection of the final model

**Figures 3A–C** show the consensus SHAP summary bar plots for the 20 highest-importance predictive features in the LightGBM, RF, and XGBoost models. Mean absolute SHAP values were calculated separately within each cross-validation training partition and then averaged across the five iterations to generate the consensus ranking. The subtype-specific bars show relative model attribution associated with each classification outcome; they should not be interpreted as independent biological or causal effects. Changes in development-stage cross-validated AUC during backward feature elimination are shown in **Figure 3D**.

**Figure 3:**
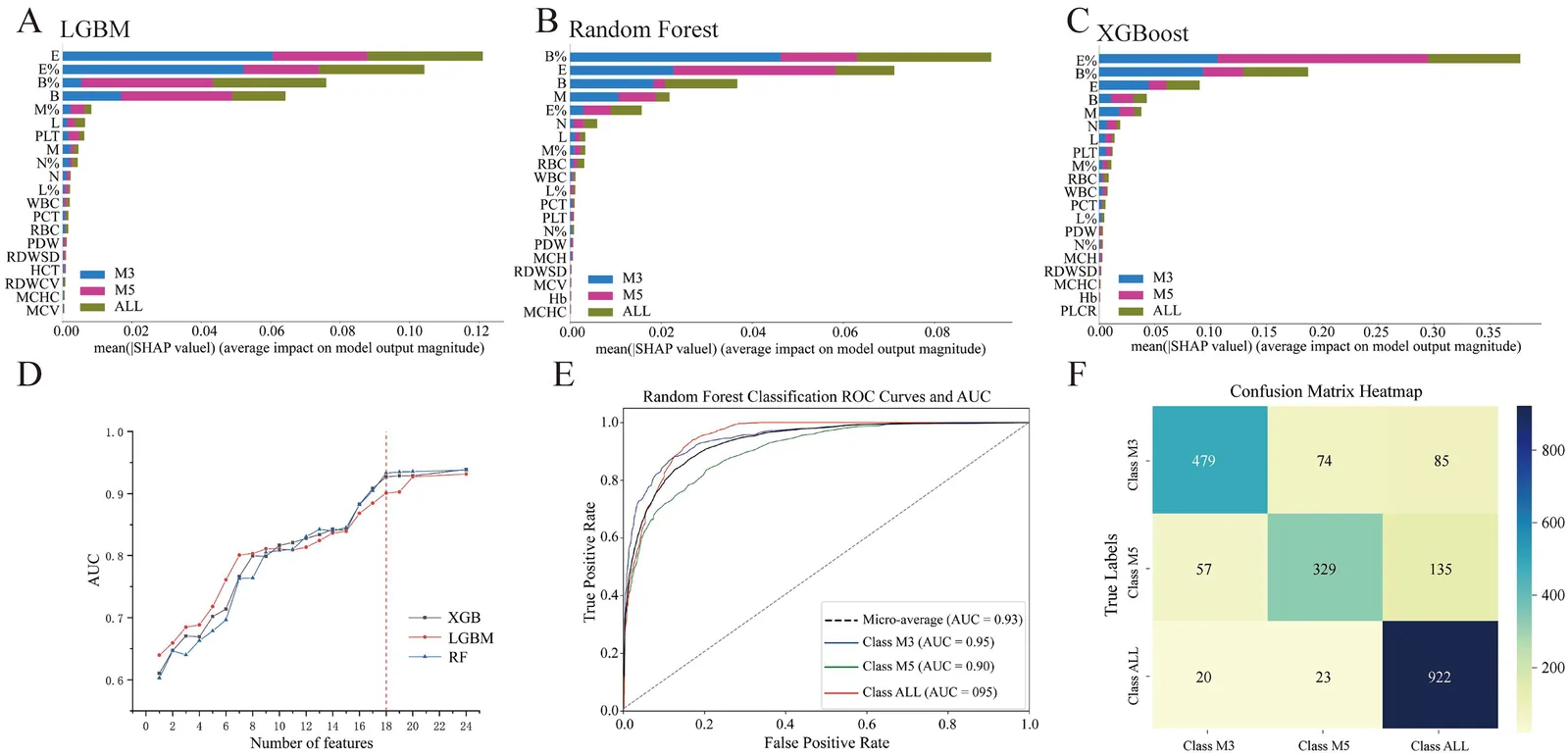
Development-stage model comparison and feature selection using five-fold stratified cross-validation. **(A–C)** Consensus SHAP summary bar plots for the LightGBM, RF, and XGBoost models. Mean absolute SHAP values were calculated separately within each cross-validation training partition and averaged across the five iterations. For each feature, bar length and color indicate relative model attribution for each leukemia subtype and should not be interpreted as a causal effect. **(D)** Development-stage cross-validated AUCs of the LightGBM, RF, and XGBoost models across different feature-set sizes. **(E)** Development-stage cross-validated receiver operating characteristic curves for the final 18-feature RF model. **(F)** Corresponding cross-validated confusion matrix.

During development-stage cross-validation, reducing the SHAP-ranked RF feature set from 24 to 17 variables was associated with a decrease in AUC (0.9378 vs. 0.9046; P < 0.05). The 18-feature RF model had an observed AUC of 0.9339 compared with 0.9378 for the 24-feature model, an absolute difference of −0.0039 (P = 0.28). The nonsignificant P value was not interpreted as evidence of equivalence or noninferiority. Considering parsimony, the small observed AUC decrease at 18 features, and the larger decrease after reduction to 17 features, the 18-feature configuration was retained as the primary model. Its development-stage cross-validated receiver operating characteristic curve and confusion matrix are shown in **Figures 3E, F**. Although the area under the precision–recall curve was slightly lower than that of the full model (**Supplementary Figure S3**), the 18-feature RF model achieved an AUC of 0.93, sensitivity of 0.78, specificity of 0.90, PPV of 0.81, NPV of 0.91, accuracy of 0.81, and F1 score of 0.79 (**Supplementary Table S5**).

During development-stage cross-validation, the LightGBM AUC decreased when the feature set was reduced to 19 or fewer variables (0.9314 vs. 0.9026; P < 0.05). For XGBoost, the corresponding decrease occurred at 18 or fewer variables (0.9390 vs. 0.9271; P < 0.05). Among the 18-feature models, RF had the highest observed cross-validated AUC (RF, 0.9339; LightGBM, 0.9010; XGBoost, 0.9271; **Supplementary Table S6**).

Accordingly, the 18-feature RF model was selected as the final development-stage configuration before any external-validation data were examined. The included variables were basophil percentage, eosinophil count, basophil count, monocyte count, eosinophil percentage, neutrophil count, lymphocyte count, monocyte percentage, RBC count, WBC count, lymphocyte percentage, PCT, PLT count, neutrophil percentage, PDW, MCH, RDW-SD, and MCV.

### Collinearity assessment and confirmation of the 18-feature model

VIF analysis identified collinearity for neutrophil percentage (VIF, 9.57) and lymphocyte percentage (VIF, 8.74); all other VIFs were below 5 (**Supplementary Table S12**). Along the consensus SHAP-ranked backward-elimination path, the 18-feature RF model had a development-stage cross-validated AUC of 0.9339 versus 0.9378 for the full 24-feature model (observed difference, −0.0039; DeLong P = 0.2756). This nonsignificant result was not interpreted as evidence of equivalence or noninferiority. Reducing the model to 17 features decreased the AUC to 0.9046 (observed difference vs. the full model, −0.0332; P = 2.07 × 10^−^¹³), and the 12-feature SHAP-ranked model had an AUC of 0.8308 (observed difference, −0.1070; P = 1.38 × 10^−90^; **Supplementary Tables S6**, **S13**). The 18-feature model was retained based on parsimony and the observed AUC trajectory, rather than on an assertion of statistical equivalence.

### External validation of the final model

After the algorithm, hyperparameters, and feature set had been fixed, the final 18-feature RF model was refitted using the complete development cohort and applied without modification to three independent external cohorts. The model achieved AUCs of 0.89 at Wanbei Coal Electric Group General Hospital, 0.87 at Tongling People’s Hospital, and 0.90 at Guangdong Second Provincial General Hospital. Receiver operating characteristic curves and confusion matrices for the external cohorts are shown in **Figures 4A–F**. Precision–recall curves were also comparable across validation cohorts (**Supplementary Figure S4**), supporting the stability of model performance in previously unseen populations.

**Figure 4:**
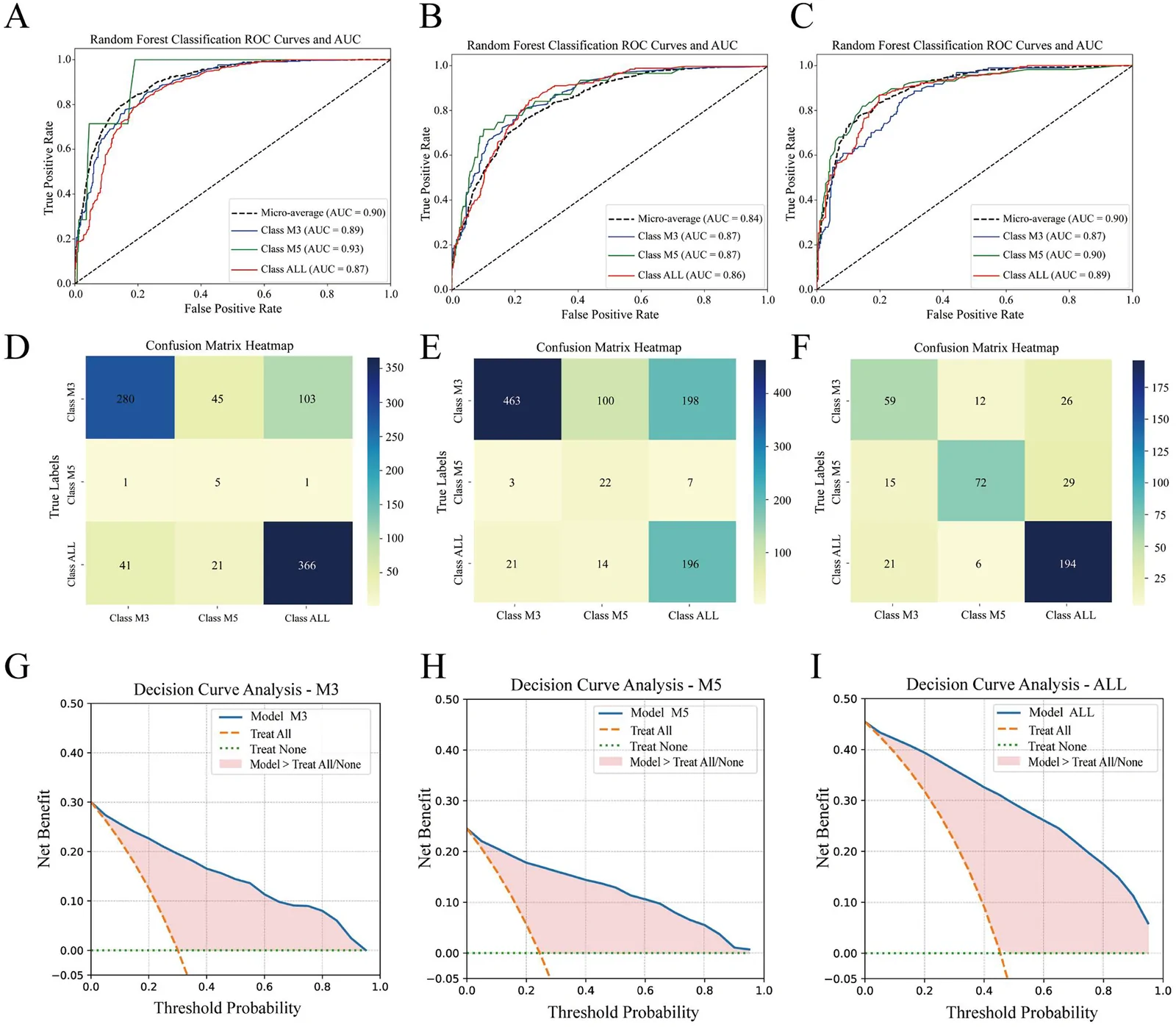
Performance of the final model in the external validation cohorts. **(A–C)** Receiver operating characteristic curves and **(D–F)** confusion matrices for the final 18-feature RF model in the three external validation cohorts. **(G–I)** Decision-curve analyses for APL/M3, AMoL/M5, and ALL classification.

#### Class-specific external-validation performance

Class-specific results revealed substantial uncertainty for AMoL/M5 in centers with few cases. In Guangdong, only 7 AMoL/M5 cases were available; the AMoL/M5 one-vs-rest AUC was 0.929 (95% CI, 0.873–0.977), sensitivity was 71.43% (95% CI, 35.89%–91.78%), and PPV was 7.04% (95% CI, 3.05%–15.45%). In Wanbei, which included 116 AMoL/M5 cases, the corresponding AUC, sensitivity, and PPV were 0.902 (95% CI, 0.867–0.934), 62.07% (95% CI, 52.99%–70.38%), and 80.00% (95% CI, 70.59%–86.96%), respectively. In Tongling, with 32 AMoL/M5 cases, the AUC was 0.874 (95% CI, 0.808–0.927), sensitivity was 68.75% (95% CI, 51.43%–82.05%), and PPV was 16.18% (95% CI, 10.93%–23.28%). Complete class-specific performance estimates for APL/M3, AMoL/M5, and ALL are provided in **Supplementary Table S14**.

Decision-curve analysis demonstrated a net clinical benefit across a range of threshold probabilities in both the development and external-validation analyses (**Figures 4G–I**; **Supplementary Figure S5**), suggesting potential utility for differentiation decisions within the restricted population already confirmed to have APL/M3, AMoL/M5, or ALL.

### Calibration of predicted probabilities

In the development cohort, the calibration intercepts were close to zero for APL/M3 (−0.023), AMoL/M5 (−0.001), and ALL (0.022), whereas the corresponding slopes were 1.570, 1.619, and 1.820. The class-specific Brier scores were 0.081, 0.100, and 0.086, respectively, and the overall multiclass Brier score was 0.267. In Wanbei, the three intercepts ranged from −0.281 to 0.152 and the overall multiclass Brier score was 0.359. More pronounced center-specific intercept shifts were observed in Tongling (APL/M3, 2.031; AMoL/M5, −2.335; ALL, −1.076; overall Brier score, 0.452) and Guangdong (APL/M3, 0.894; AMoL/M5, −3.428; ALL, 0.119; overall Brier score, 0.356), consistent with marked differences in subtype prevalence and case mix. The Guangdong AMoL/M5 estimates were particularly imprecise because only seven reference-standard cases were available. Calibration plots and complete estimates with 95% confidence intervals are presented in **Supplementary Figure S6** and **Supplementary Table S15**.

#### APL-prioritized alert performance

Under the default maximum-probability classification rule, the APL/M3-specific AUC was 0.947, sensitivity was 0.7508, and PPV was 0.8615 (**Supplementary Table S5**). For the clinically oriented alert analysis, the highest threshold achieving at least 99% APL sensitivity in the development cohort was 0.0966. At this threshold, development-cohort sensitivity was 99.02% (95% CI, 98.49%–99.37%) and PPV was 54.34% (95% CI, 52.73%–55.93%). When the threshold was applied unchanged, sensitivity was 97.66% in Guangdong, 97.94% in Wanbei, and 96.32% in Tongling; the corresponding PPVs were 66.24%, 35.58%, and 84.25%, respectively. The numbers of APL cases not flagged were 10, 2, and 28, respectively (**Supplementary Table S11**). These results demonstrate the expected trade-off of a high-sensitivity alert: substantially fewer missed APL cases at the cost of more false-positive alerts, with PPV varying according to cohort case mix and APL prevalence.

### Web-based model implementation

To support clinical evaluation, the final model was implemented as a web-based application (**Figure 5**). Users enter the 18 required hematological parameters, after which the application displays the predicted probability of each subtype and highlights the class with the highest probability. The application is publicly accessible at https://acute-leukaemias-4gnpgqp9nppu2of84cggyr.streamlit.app/. Its intended use is limited to adjunctive differentiation among APL/M3, AMoL/M5, and ALL in patients already confirmed to belong to one of these three diagnostic categories; it is not a general acute-leukemia screening tool. The revised interface implements the APL alert independently of the maximum-probability classification. In the example shown in **Figure 5**, ALL remains the maximum-probability class (0.680), but the APL probability (0.280) exceeds the prespecified threshold of 0.0966 and therefore generates an explicit APL alert with recommendations for urgent smear review, coagulation assessment, PML::RARA testing, and specialist hematology assessment. The interface also displays a permanent warning that the model cannot exclude APL or replace morphological and molecular confirmation.

**Figure 5:**
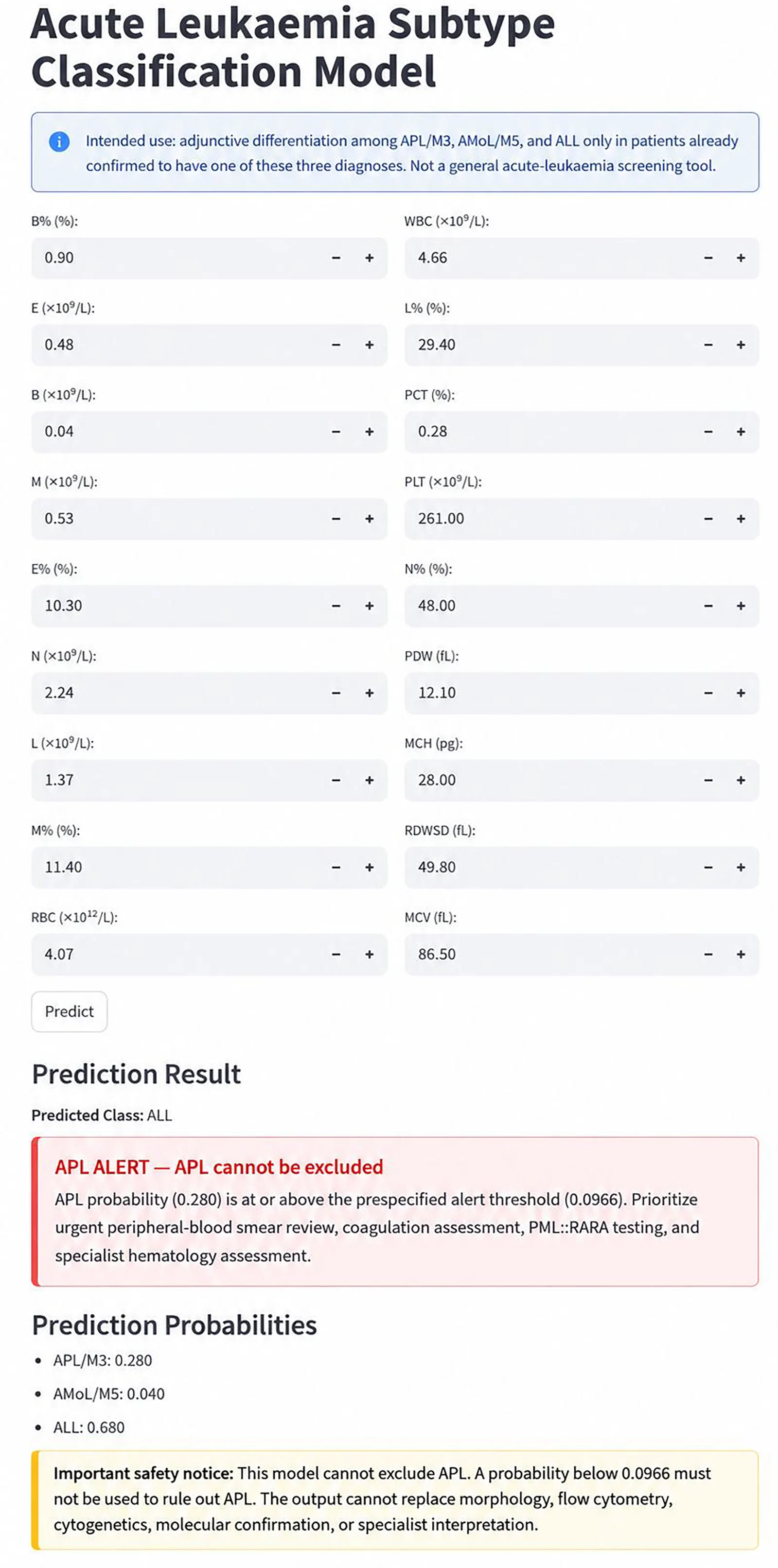
Revised web-based application implementing the APL-prioritized alert. In the illustrated example, ALL has the highest predicted probability (0.680), whereas the APL/M3 probability is 0.280. Because 0.280 exceeds the prespecified APL alert threshold of 0.0966, the application displays an explicit warning that APL cannot be excluded and prioritizes urgent peripheral-blood smear review, coagulation assessment, PML::RARA testing, and specialist hematology assessment. The permanent safety notice emphasizes that the application cannot exclude APL or replace morphological and molecular confirmation.

## Discussion

The increasing use of artificial intelligence and machine learning in hematology has created opportunities for computer-assisted leukemia detection and classification. However, many published approaches rely on morphological image analysis and are limited by small sample sizes, variable image quality, and the absence of independent external validation, all of which may restrict clinical generalizability (22).

In contrast, the present study used routinely available hematological parameters rather than cellular images to classify APL/M3, AMoL/M5, and ALL. The final RF model maintained good discrimination across three independent external cohorts within this preselected three-category diagnostic population. These results do not establish performance in unselected patients with suspected acute leukemia or in patients with other hematological or non-malignant conditions. The approach is intended to complement, rather than replace, morphological and immunophenotypic assessment.

The external cohorts were markedly imbalanced with respect to AMoL/M5, particularly in Guangdong (n = 7) and Tongling (n = 32). Consequently, overall or macro-averaged metrics should not be interpreted as evidence of equally reliable performance for every subtype. Although the AMoL/M5 one-vs-rest AUCs were 0.874–0.929, the sensitivity confidence intervals were wide and PPV was only 7.04% in Guangdong and 16.18% in Tongling. These findings reflect both limited sample size and center-specific disease prevalence and indicate that the external generalizability of AMoL/M5 classification remains uncertain.

The calibration analysis further qualifies the interpretation of the model output. Although calibration intercepts were near zero in the development cohort, slopes greater than 1 indicated that the cross-validated predictions were somewhat under-dispersed. External calibration varied by center, with substantial intercept shifts in Tongling and Guangdong that were consistent with their different subtype prevalences and case mix. Accordingly, the displayed numerical outputs should not be interpreted as universally transportable absolute diagnostic probabilities. The APL cutoff of 0.0966 is an operating threshold selected to prioritize sensitivity within this restricted three-category population, rather than a statement that an individual has a 9.66% absolute probability of APL. Before probabilities are used quantitatively in a new institution, prospective local validation and, if necessary, recalibration are required. The very small number of AMoL/M5 cases in Guangdong also makes its class-specific calibration estimate unstable.

In institutions with established automated complete-blood-count review rules and continuous access to experienced morphological assessment, the present model has not been shown to provide a time or efficiency advantage over standard microscopy. The intended users are hematologists and laboratory professionals who are evaluating patients already confirmed to have APL/M3, AMoL/M5, or ALL and require adjunctive support for differentiation among these three categories. The model has not been evaluated in the broader population encountered by emergency or general physicians and should not be used as a stand-alone triage or screening tool in that setting. Because other AML subtypes, mixed-phenotype acute leukemia, other hematological malignancies, and non-malignant mimics were not represented, every input will be assigned to one of the three modeled categories even when the true diagnosis lies outside them. The model is not intended to establish a definitive diagnosis or to replace specialist assessment, morphology, flow cytometry, cytogenetics, or molecular testing.

A direct comparison with morphological screening could not be performed because the retrospective dataset did not contain standardized, independently recorded morphology-screening classifications or comparable turnaround-time measurements. Accordingly, we do not claim that the model is faster, more accurate, or more efficient than expert morphological screening. A prospective workflow study comparing automated review rules, model-assisted triage, and routine microscopy is required to quantify incremental clinical value.

Machine-learning methods can capture complex, nonlinear relationships within high-dimensional clinical data (23). Combining electronic medical record data with appropriate algorithms may therefore support the development of practical clinical prediction tools. Among the ten algorithms evaluated in this study, RF provided the best balance of discrimination, stability, and parsimony. RF combines multiple decision trees and aggregates their predictions, an approach that has performed well in medical classification and risk-prediction tasks (24, 25).

Although correlated predictors can complicate claims of interpretability, their impact depends on the modeling method. Random forests do not estimate regression coefficients and use bootstrap aggregation and random predictor subsampling, making their predictive performance relatively robust to multicollinearity. Correlated variables may still compete for similar splits and divide feature-importance or SHAP attribution, so the importance assigned to an individual percentage or absolute count should not be interpreted as an independent causal effect. Our VIF analysis confirmed collinearity for neutrophil and lymphocyte percentages. However, percentages describe relative cellular composition, whereas absolute counts describe circulating cell burden, and random forests can use their nonlinear interactions differently. The observed AUC trajectory showed only a small decrease from the 24-feature to the 18-feature model, followed by larger decreases for the 17- and 12-feature SHAP-ranked models. The nonsignificant DeLong result for the 18-feature comparison was not interpreted as evidence of equivalence or noninferiority. We therefore retained the 18-feature model based on parsimony and the observed performance trajectory while tempering the interpretation of individual SHAP values. Because these predictors are automatically generated by routine hematology analyzers, their retention does not require additional specimen collection or laboratory testing, although automated transfer from the laboratory information system would be preferable to manual entry.

Our study is closely related to, but differs from, the AI-PAL model developed by Alcazer et al. (20). AI-PAL used a ten-variable XGBoost model incorporating age, complete blood count indices, lactate dehydrogenase, prothrombin activity, and fibrinogen to distinguish APL, non-APL AML, and ALL in 1,410 patients from six French hospitals. In comparison, our model was developed in 9,398 patients from four Chinese hospitals, used only complete blood count parameters, and specifically distinguished AMoL/M5 from APL/M3 and ALL. These differences in target populations, disease categories, predictor availability, and performance-estimation methods preclude a valid direct numerical comparison. Therefore, the present model should be viewed as complementary to AI-PAL rather than superior to it. AI-PAL offers a more parsimonious multimodal laboratory model, whereas our findings evaluate the discriminatory information available from complete blood counts alone in a large East Asian multicenter cohort.

The restriction to complete blood count parameters represents both a deliberate design choice and a limitation. Biochemical and coagulation variables, including lactate dehydrogenase, prothrombin time or activity, and fibrinogen, provide clinically relevant information and were included among the predictors in AI-PAL (20). Their inclusion could plausibly improve discrimination and reduce misclassification, particularly for APL, in which coagulopathy is a defining clinical concern. Accordingly, our model should be interpreted as a CBC-only adjunctive classifier for the restricted three-category population rather than a general triage tool for suspected acute leukemia or the best achievable multimodal laboratory classifier. Future prospective studies should directly compare the CBC-only model with an extended model incorporating biochemical and coagulation parameters, while evaluating the trade-off between predictive performance, data availability, and implementation burden.

Misclassification is unavoidable in a model based solely on routine hematological parameters. First, many of these variables are nonspecific and may be altered by infection, inflammation, allergy, parasitic disease, medication, or other hematologic conditions. Second, overlapping phenotypes among leukemia subtypes and imbalances in subtype prevalence across centers may limit discrimination. Future studies should assess domain shift explicitly, include common mimicking conditions and additional leukemia subtypes, and evaluate whether multimodal or multitask approaches improve robustness.

The three leukemia subtypes do not carry symmetric consequences of misclassification. In particular, failure to recognize APL may delay urgent assessment and management of potentially life-threatening coagulopathy. We therefore supplemented the original three-class output with an APL-prioritized alert threshold selected to achieve at least 99% sensitivity in the development cohort. The locked threshold retained high APL sensitivity across all three external cohorts (96.32%–97.94%), although PPV varied substantially (35.58%–84.25%), consistent with differences in disease prevalence and case mix. This alert is intended to prioritize urgent smear review, coagulation testing, PML::RARA confirmation, and specialist assessment; it is not a diagnostic threshold and must not independently trigger or withhold treatment. Moreover, even this high-sensitivity strategy missed some APL cases, so a probability below 0.0966 must not be used to rule out APL when clinical or morphological findings remain suspicious. This independent alert logic has now been implemented in the web application: an APL alert is displayed whenever the predicted APL probability is at or above 0.0966, even if AMoL/M5 or ALL has the highest predicted probability. The interface also displays an explicit warning that the model cannot exclude APL and cannot replace morphological or molecular confirmation.

This study has several limitations. First, both model development and external validation were restricted to Chinese cohorts; multinational validation is required before broader application. Second, the model included only patients already confirmed to have APL/M3, AMoL/M5, or ALL. Other AML subtypes, mixed-phenotype acute leukemia, other hematological malignancies, and non-malignant mimics were not represented and would necessarily be assigned to one of the three modeled categories. Therefore, the model should not be used to screen or rule out acute leukemia in an unselected clinical population. Third, the retrospective design and center-specific differences in disease prevalence may introduce selection and spectrum bias. The very small AMoL/M5 samples in the Guangdong and Tongling cohorts produced imprecise class-specific estimates and preclude strong conclusions regarding external generalizability for this subtype. Fourth, standardized morphological screening results and turnaround times were unavailable, precluding a head-to-head comparison with routine microscopy or estimation of time saved. Fifth, because candidate-algorithm comparison and feature selection were performed within the same development cohort, the development-stage cross-validated performance estimates may be optimistic owing to selection-related overfitting and should not be interpreted as independent validation results. In contrast, the three external cohorts were not used for preprocessing, hyperparameter tuning, feature ranking, feature elimination, algorithm comparison, or model selection and therefore provide independent evaluations of the locked final model. Finally, the model provides only preliminary decision support within the restricted three-category population and cannot replace confirmation by morphology, flow cytometry, cytogenetics, or molecular testing (21). Because eligibility was restricted to *de novo* disease, the findings should not be extrapolated to patients receiving treatment or those in remission or relapse. Low-confidence or clinically discordant predictions should be interpreted cautiously and verified using standard diagnostic procedures.

In conclusion, we developed and externally validated an interpretable RF model that differentiates APL/M3, AMoL/M5, and ALL using 18 routinely available hematological parameters in patients already confirmed to have one of these three diagnoses. The model achieved a development-stage five-fold cross-validated AUC of 0.93 and external AUCs of 0.87–0.90 across three independent hospitals. A web-based implementation was created to support prospective evaluation within this restricted diagnostic population. These findings suggest that routine blood-test data may aid adjunctive differentiation and prioritization of subtype-specific confirmatory testing among the three modeled categories, but the model should not be used for general acute-leukemia screening. Prospective, multinational, and real-world implementation studies are required before clinical adoption.

## Data Availability

The original contributions presented in the study are included in the article/**Supplementary Material**. Further inquiries can be directed to the corresponding authors.
