# Supplementary material for: Multicenter validation of an explainable machine-learning model for APL, AMoL, and ALL
Source: Front Oncol. 2026 Sep 29;16:1939329. doi: 10.3389/fonc.2026.1939329 (PMC13657969; doi:10.3389/fonc.2026.1939329)
Supplement: Supplementary file 1 [file SupplementaryFile1.docx]

**Table S1. Clinical characteristics of patients at the The Affiliated Guangdong Second Provincial General Hospital of Jinan University.**

| **Characteristic** | **group** | | | **p-value** |
| --- | --- | --- | --- | --- |
|  | M3  N = 428 | M5  N = 7 | ALL  N = 428 |  |
| Age | 47 (29, 54) | 55 (39, 63) | 42 (24, 56) | 0.127 |
| Sex, n (%) |  |  |  | 0.432 |
| Male | 198 (46.3%) | 5 (71.4%) | 203 (47.4%) |  |
| Female | 230 (53.7%) | 2 (28.6%) | 225 (52.6%) |  |
| Basophil(%) | 0.20 (0.00, 0.50) | 0.10 (0.00, 0.60) | 0.04 (0.00, 0.40) | <0.001 |
| Eosinophil | 0.07 (0.02, 0.13) | 0.00 (0.00, 0.25) | 0.00 (0.00, 0.03) | <0.001 |
| Basophil | 0.010 (0.010, 0.030) | 0.010 (0.000, 0.030) | 0.010 (0.000, 0.020) | <0.001 |
| Monocyte | 0.34 (0.20, 0.51) | 0.27 (0.04, 0.64) | 0.24 (0.03, 0.53) | <0.001 |
| Eosinophil(%) | 1.20 (0.08, 2.85) | 0.04 (0.00, 1.60) | 0.04 (0.00, 0.52) | <0.001 |
| Neutrophil | 2.7 (1.6, 4.4) | 0.6 (0.1, 7.6) | 1.9 (0.4, 4.0) | <0.001 |
| Lymphocyte | 1.25 (0.82, 1.84) | 0.93 (0.45, 1.15) | 0.66 (0.30, 1.17) | <0.001 |
| Monocyte% | 6 (1, 9) | 7 (4, 11) | 6 (1, 12) | 0.663 |
| RBC, 1012/L | 3.65 (2.67, 4.23) | 2.63 (1.80, 3.80) | 2.82 (2.27, 3.47) | <0.001 |
| WBC, 109/L | 5 (3, 7) | 2 (1, 11) | 3 (1, 7) | <0.001 |
| Lymphocyte(%) | 27 (10, 37) | 25 (10, 72) | 24 (6, 46) | 0.782 |
| PCT(%) | 0.18 (0.13, 0.25) | 0.07 (0.01, 0.13) | 0.14 (0.08, 0.17) | <0.001 |
| PLT, 109/L | 163 (71, 221) | 60 (17, 107) | 73 (24, 172) | <0.001 |
| Neutrophil(%) | 54 (36, 66) | 64 (25, 79) | 53 (13, 74) | 0.812 |
| PDW, fL | 12.80 (11.30, 15.70) | 10.80 (10.00, 13.30) | 12.00 (11.20, 14.50) | 0.003 |
| MCH, pg | 31.30 (29.85, 32.70) | 32.00 (31.20, 32.40) | 29.90 (28.55, 32.70) | <0.001 |
| RDW-SD, fL | 50 (45, 56) | 52 (41, 55) | 48 (43, 58) | 0.384 |
| MCV, fL | 95 (90, 99) | 95 (86, 102) | 88 (85, 101) | <0.001 |

Continuous values were presented as median [interquartile range]. Categorical values were presented as number (percentage).

WBC, white blood cell; Neutrophil; Neutrophil%; Eosinophil; Eosinophil%; Basophil; Basophil%; Monocyte; Monocyte%; Lymphocyte; Lymphocyte%; RBC, red blood cell; MCV, mean corpuscular volume; MCH, mean corpuscular hemoglobin; RDW-SD, red blood cell distribution width - standard deviation; PLT, platelet count; PCT, platelet accumulation; PDW, platelet distribution width.

**Table S2. Clinical characteristics of patients at TongLing People's Hospital.**

| **Characteristic** | **group** | | | **p-value** |
| --- | --- | --- | --- | --- |
|  | M3  N = 761 | M5  N = 32 | ALL  N = 231 |  |
| Age | 45 (28, 55) | 41(37, 56) | 36(22, 55) | 0.105 |
| Sex, n (%) |  |  |  | 0.051 |
| Male | 336 (44.2%) | 21 (65.6%) | 100 (43.3%) |  |
| Female | 425 (55.8%) | 11 (34.4%) | 131 (56.7%) |  |
| Basophil(%) | 0.20 (0.00, 0.50) | 0.15 (0.02, 0.35) | 0.20 (0.00, 0.50) | 0.352 |
| Eosinophil | 0.06 (0.02, 0.13) | 0.01 (0.00, 0.03) | 0.01 (0.00, 0.06) | <0.001 |
| Basophil | 0.010 (0.010, 0.030) | 0.010 (0.000, 0.010) | 0.010 (0.000, 0.020) | 0.009 |
| Monocyte | 0.32 (0.17, 0.49) | 0.24 (0.03, 0.44) | 0.24 (0.03, 0.50) | <0.001 |
| Eosinophil(%) | 1.00 (0.10, 2.60) | 0.12 (0.00, 0.95) | 0.06 (0.00, 1.40) | <0.001 |
| Neutrophil | 2.5 (1.5, 4.2) | 2.0 (0.1, 4.0) | 2.0 (0.7, 3.8) | <0.001 |
| Lymphocyte | 1.23 (0.78, 1.86) | 0.72 (0.33, 0.81) | 0.76 (0.35, 1.22) | <0.001 |
| Monocyte% | 6.3 (1.3, 9.5) | 6.6 (1.9, 9.2) | 6.0 (1.1, 11.1) | 0.994 |
| RBC, 1012/L | 3.61 (2.69, 4.15) | 2.77 (2.17, 3.51) | 3.08 (2.45, 3.63) | <0.001 |
| WBC, 109/L | 5 (3, 7) | 2 (0, 7) | 3 (2, 6) | <0.001 |
| Lymphocyte(%) | 27 (12, 37) | 26 (19, 58) | 21 (9, 41) | 0.302 |
| PCT(%) | 0.18 (0.12, 0.24) | 0.14 (0.05, 0.14) | 0.14 (0.10, 0.17) | <0.001 |
| PLT, 109/L | 158 (77, 219) | 33 (17, 84) | 84 (28, 160) | <0.001 |
| Neutrophil(%) | 55 (36, 67) | 60 (22, 69) | 60 (28, 76) | 0.029 |
| PDW, fL | 12.8 (11.0, 15.8) | 12.0 (11.7, 16.7) | 12.0 (11.3, 14.2) | 0.040 |
| MCH, pg | 31.30 (29.90, 32.50) | 30.75 (29.95, 31.95) | 30.10 (28.60, 32.90) | <0.001 |
| RDW-SD, fL | 50 (45, 56) | 49 (44, 56) | 48 (42, 60) | 0.354 |
| MCV, fL | 94 (90, 98) | 92 (88, 96) | 88 (85, 101) | <0.001 |

Continuous values were presented as median [interquartile range]. Categorical values were presented as number (percentage).

WBC, white blood cell; Neutrophil; Neutrophil%; Eosinophil; Eosinophil%; Basophil; Basophil%; Monocyte; Monocyte%; Lymphocyte; Lymphocyte%; RBC, red blood cell; MCV, mean corpuscular volume; MCH, mean corpuscular hemoglobin; RDW-SD, red blood cell distribution width - standard deviation; PLT, platelet count; PCT, platelet accumulation; PDW, platelet distribution width.

**Table S3. Clinical characteristics of patients at Wanbei Coal Electric Group General Hospital**

| **Characteristic** | **group** | | | **p-value** |
| --- | --- | --- | --- | --- |
|  | M3  N = 97 | M5  N = 116 | ALL  N = 221 |  |
| Age | 47 (29, 54) | 50 (34, 56) | 43 (28, 56) | 0.409 |
| Sex, n (%) |  |  |  | 0.215 |
| Male | 56 (57.7%) | 53 (45.7%) | 114 (51.6%) |  |
| Female | 41 (42.3%) | 63 (54.3%) | 107 (48.4%) |  |
| Basophil(%) | 0.30 (0.00, 0.40) | 0.04 (0.00, 0.40) | 0.10 (0.00, 0.40) | 0.294 |
| Eosinophil | 0.07 (0.01, 0.14) | 0.01 (0.00, 0.07) | 0.01 (0.00, 0.05) | <0.001 |
| Basophil | 0.010 (0.010, 0.030) | 0.005 (0.000, 0.010) | 0.010 (0.000, 0.020) | 0.001 |
| Monocyte | 0.33 (0.22, 0.53) | 0.24 (0.03, 0.52) | 0.24 (0.05, 0.55) | 0.031 |
| Eosinophil(%) | 1.30 (0.10, 2.80) | 0.30 (0.00, 1.95) | 0.04 (0.00, 0.90) | <0.001 |
| Neutrophil | 2.7 (1.5, 4.1) | 1.5 (0.2, 3.0) | 2.0 (0.7, 4.2) | <0.001 |
| Lymphocyte | 1.22 (0.76, 1.96) | 0.76 (0.32, 1.14) | 0.70 (0.31, 1.10) | <0.001 |
| Monocyte% | 7 (3, 9) | 7 (3, 16) | 7 (2, 13) | 0.199 |
| RBC, 1012/L | 3.44 (2.85, 4.14) | 2.88 (1.99, 3.59) | 2.89 (2.44, 3.48) | <0.001 |
| WBC, 109/L | 5 (3, 7) | 3 (1, 5) | 3 (1, 6) | <0.001 |
| Lymphocyte(%) | 27 (16, 35) | 30 (15, 54) | 21 (6, 40) | 0.001 |
| PCT(%) | 0.16 (0.10, 0.24) | 0.12 (0.04, 0.14) | 0.14 (0.09, 0.18) | <0.001 |
| PLT, 109/L | 146 (69, 214) | 51 (18, 103) | 78 (26, 168) | <0.001 |
| Neutrophil(%) | 58 (42, 69) | 54 (13, 68) | 60 (33, 78) | 0.055 |
| PDW, fL | 12.80 (11.65, 15.45) | 12.00 (10.80, 13.80) | 12.00 (10.60, 13.30) | <0.001 |
| MCH, pg | 31.30 (29.90, 32.60) | 31.55 (30.40, 32.80) | 29.90 (28.50, 32.60) | <0.001 |
| RDW-SD, fL | 50 (46, 55) | 51 (45, 62) | 49 (42, 63) | 0.313 |
| MCV, fL | 95 (91, 98) | 94 (90, 99) | 87 (85, 102) | <0.001 |

Continuous values were presented as median [interquartile range]. Categorical values were presented as number (percentage).

WBC, white blood cell; Neutrophil; Neutrophil%; Eosinophil; Eosinophil%; Basophil; Basophil%; Monocyte; Monocyte%; Lymphocyte; Lymphocyte%; RBC, red blood cell; MCV, mean corpuscular volume; MCH, mean corpuscular hemoglobin; RDW-SD, red blood cell distribution width - standard deviation; PLT, platelet count; PCT, platelet accumulation; PDW, platelet distribution width.

**Table S4. Pairwise DeLong comparisons of the ten candidate models based on development-stage five-fold cross-validated predictions.**

| **Model 1** | **AUC 1** | **Model 2** | **AUC 2** | **P-value** |
| --- | --- | --- | --- | --- |
| XGB | 0.9390 | RF | 0.9378 | 0.77 |
| XGB | 0.9390 | Adaboost | 0.8456 | <0.01 |
| XGB | 0.9390 | DT | 0.8454 | <0.01 |
| XGB | 0.9390 | GB | 0.9231 | <0.01 |
| XGB | 0.9390 | KNN | 0.8183 | <0.01 |
| XGB | 0.9390 | LGBM | 0.9314 | 0.07 |
| XGB | 0.9390 | LR | 0.6847 | <0.01 |
| XGB | 0.9390 | MLP | 0.6995 | <0.01 |
| XGB | 0.9390 | SVM | 0.824 | <0.01 |
| RF | 0.9378 | Adaboost | 0.8456 | <0.01 |
| RF | 0.9378 | DT | 0.8454 | <0.01 |
| RF | 0.9378 | GB | 0.9231 | <0.01 |
| RF | 0.9378 | KNN | 0.8183 | <0.01 |
| RF | 0.9378 | LGBM | 0.9314 | 0.12 |
| RF | 0.9378 | LR | 0.6847 | <0.01 |
| RF | 0.9378 | MLP | 0.6995 | <0.01 |
| RF | 0.9378 | SVM | 0.8240 | <0.01 |
| Adaboost | 0.8456 | DT | 0.8454 | 0.97 |
| Adaboost | 0.8456 | GB | 0.9231 | <0.01 |
| Adaboost | 0.8456 | KNN | 0.8183 | <0.01 |
| Adaboost | 0.8456 | LGBM | 0.9314 | <0.01 |
| Adaboost | 0.8456 | LR | 0.6847 | <0.01 |
| Adaboost | 0.8456 | MLP | 0.6995 | <0.01 |
| Adaboost | 0.8456 | SVM | 0.824 | <0.01 |
| DT | 0.8454 | GB | 0.9231 | <0.01 |
| DT | 0.8454 | KNN | 0.8183 | <0.01 |
| DT | 0.8454 | LGBM | 0.9314 | <0.01 |
| DT | 0.8454 | LR | 0.6847 | <0.01 |
| DT | 0.8454 | MLP | 0.6995 | <0.01 |
| DT | 0.8454 | SVM | 0.8240 | <0.01 |
| GB | 0.9231 | KNN | 0.8183 | <0.01 |
| GB | 0.9231 | LGBM | 0.9314 | 0.06 |
| GB | 0.9231 | LR | 0.6847 | <0.01 |
| GB | 0.9231 | MLP | 0.6995 | <0.01 |
| GB | 0.9231 | SVM | 0.8240 | <0.01 |
| KNN | 0.8183 | LGBM | 0.9314 | <0.01 |
| KNN | 0.8183 | LR | 0.6847 | <0.01 |
| KNN | 0.8183 | MLP | 0.6995 | <0.01 |
| KNN | 0.8183 | SVM | 0.8240 | 0.38 |
| LGBM | 0.9314 | LR | 0.6847 | <0.01 |
| LGBM | 0.9314 | MLP | 0.6995 | <0.01 |
| LGBM | 0.9314 | SVM | 0.8240 | <0.01 |
| LR | 0.6847 | MLP | 0.6995 | 0.06 |
| LR | 0.6847 | SVM | 0.8240 | <0.01 |
| MLP | 0.6995 | SVM | 0.8240 | <0.01 |

All model comparisons in this table were performed within the development cohort. The three external-validation cohorts were not used for algorithm comparison or model selection. A nonsignificant P value indicates that no statistically significant AUC difference was detected and does not establish equivalence or noninferiority. XGB, XGBoost; RF, random forest; DT, decision tree; GB, gradient boosting; KNN, k-nearest neighbors; AdaBoost, adaptive boosting; SVM, support vector machine; LR, logistic regression; LGBM, light gradient boosting machine; MLP, multilayer perceptron.

**Table S5. Class-specific five-fold cross-validated performance of the 18-feature RF model in the development cohort.**

| **RF** | **APL/M3** | **AMoL/M5** | **ALL** | **Average** |
| --- | --- | --- | --- | --- |
| AUC | 0.947 | 0.9048 | 0.9449 | 0.93 |
| Sensitivity | 0.7508 | 0.6315 | 0.9554 | 0.78 |
| Specificity | 0.9482 | 0.9395 | 0.8102 | 0.90 |
| PPV | 0.8615 | 0.7723 | 0.8074 | 0.81 |
| NPV | 0.8986 | 0.8869 | 0.9562 | 0.91 |
| Accuracy | 0.86 | 0.77 | 0.81 | 0.81 |
| F1 score | 0.8 | 0.69 | 0.88 | 0.79 |

**Table S6. Development-stage five-fold cross-validated AUCs of XGBoost, LightGBM, and RF models across different feature-set sizes.**

| **Number of parameters** | **AUC(XGB)** | **AUC(LGBM)** | **AUC(RF)** |
| --- | --- | --- | --- |
| 1 | 0.6106 | 0.6397 | 0.6029 |
| 2 | 0.6473 | 0.6593 | 0.6466 |
| 3 | 0.6704 | 0.6848 | 0.6401 |
| 4 | 0.6693 | 0.6883 | 0.6631 |
| 5 | 0.7019 | 0.7178 | 0.6787 |
| 6 | 0.7137 | 0.7608 | 0.6965 |
| 7 | 0.7663 | 0.8005 | 0.7628 |
| 8 | 0.7996 | 0.8033 | 0.7639 |
| 9 | 0.7986 | 0.8113 | 0.8052 |
| 10 | 0.8169 | 0.8111 | 0.8087 |
| 11 | 0.8213 | 0.8094 | 0.8103 |
| 12 | 0.8282 | 0.8139 | 0.8308 |
| 13 | 0.8339 | 0.8245 | 0.8425 |
| 14 | 0.8428 | 0.8364 | 0.8397 |
| 15 | 0.8408 | 0.8393 | 0.8454 |
| 16 | 0.8828 | 0.8683 | 0.8824 |
| 17 | 0.9084 | 0.8847 | 0.9046 |
| 18 | 0.9271 | 0.901 | 0.9339 |
| 19 | 0.9287 | 0.9026 | 0.9348 |
| 20 | 0.9287 | 0.9271 | 0.9356 |
| 24 | 0.939 | 0.9314 | 0.9378 |

**Table S7. Changes in macro-averaged AUC after z-score standardization in five-fold stratified cross-validation.**

| **Model** | **ΔAUC** |
| --- | --- |
| MLP | +0.1091 |
| SVM | +0.0786 |
| KNN | +0.0418 |
| LGBM | +0.0011 |
| RF | +0.0002 |
| DT | +0.0002 |
| GB | +0.0000 |
| AdaBoost | +0.0000 |
| XGB | 0.0000 |
| LR | −0.0029 |

ΔAUC was calculated as the macro-averaged one-vs-rest AUC obtained after z-score standardization minus that obtained without standardization. Positive values indicate improved performance after standardization. Standardization parameters were estimated exclusively from the training partition within each cross-validation fold. AUC, area under the receiver operating characteristic curve; LR, logistic regression; MLP, multilayer perceptron; SVM, support vector machine; KNN, k-nearest neighbors; LGBM, light gradient boosting machine; RF, random forest; DT, decision tree; GB, gradient boosting; XGB, extreme gradient boosting.

**Table S8. Missing-data rates for the 24 candidate hematological parameters in the development cohort (N = 7,077).**

| **Variable** | **Missing, n (%)** |
| --- | --- |
| WBC | 7 (0.10%) |
| Neutrophil | 417 (5.89%) |
| Neutrophil% | 418 (5.91%) |
| Eosinophil | 206 (2.91%) |
| Eosinophil% | 207 (2.93%) |
| Basophil | 204 (2.88%) |
| Basophil% | 209 (2.95%) |
| Lymphocyte | 364 (5.14%) |
| Lymphocyte% | 361 (5.10%) |
| Monocyte | 366 (5.17%) |
| Monocyte% | 363 (5.13%) |
| RBC | 1 (0.01%) |
| RDW-CV | 84 (1.19%) |
| RDW-SD | 87 (1.23%) |
| HGB | 3 (0.04%) |
| HCT | 1 (0.01%) |
| MCH | 1 (0.01%) |
| MCV | 13 (0.18%) |
| MCHC | 6 (0.08%) |
| PLT | 3 (0.04%) |
| PCT | 1,177 (16.63%) |
| PDW | 1,185 (16.74%) |
| MPV | 1,182 (16.70%) |
| P-LCR | 1,176 (16.62%) |

WBC, white blood cell count; RBC, red blood cell count; RDW-CV, red cell distribution width coefficient of variation; RDW-SD, red cell distribution width standard deviation; HGB, hemoglobin; HCT, hematocrit; MCH, mean corpuscular hemoglobin; MCV, mean corpuscular volume; MCHC, mean corpuscular hemoglobin concentration; PLT, platelet count; PCT, plateletcrit; PDW, platelet distribution width; MPV, mean platelet volume; P-LCR, platelet large-cell ratio.

**Table S9. Sensitivity analysis comparing imputation methods for the final 18-feature random forest model.**

| **Imputation method** | **Macro-averaged AUC** | ΔAUC vs. mean |
| --- | --- | --- |
| Mean | 0.9415 | Reference |
| Median | 0.9414 | −0.0001 |
| Random forest | 0.9399 | −0.0016 |

AUCs were obtained using five-fold stratified cross-validation. Within each fold, the imputation procedure was fitted exclusively to the training partition and applied to the corresponding validation partition. ΔAUC was calculated relative to mean imputation. AUC, area under the receiver operating characteristic curve.

**Table S10. Prespecified hyperparameter search spaces for the ten candidate machine-learning algorithms.**

| **Model** | **Hyperparameter search space** | **Selected hyperparameters** |
| --- | --- | --- |
| RF | n_estimators: 100, 200, 500;  max_depth: None, 10, 20;  max_features: sqrt, log2; min_samples_leaf: 1, 2, 5;  class_weight: None, balanced | n_estimators: 100; criterion: gini; max_depth: None; max_features: sqrt; min_samples_split: 2; min_samples_leaf: 1; bootstrap: True; class_weight: balanced; random_state: None |
| XGB | n_estimators: 100, 200, 300;  max_depth: 3, 5, 7;  learning_rate: 0.03, 0.1, 0.2;  subsample: 0.8, 1.0;  colsample_bytree: 0.8, 1.0 | n_estimators:200  max_depth: 3  learning_rate:0.1  subsample: 0.8  colsample_bytree:1.0 |
| AdaBoost | n_estimators: 50, 100, 200;  learning_rate: 0.05, 0.1, 0.5, 1.0 | n_estimators: 50  learning_rate: 1.0 |
| GB | n_estimators: 100, 200;  learning_rate: 0.05, 0.1;  max_depth: 2, 3, 5;  subsample: 0.8, 1.0 | n_estimators: 100,  learning_rate: 0.05  max_depth: 2  subsample:1.0 |
| LGBM | n_estimators: 100, 200, 300;  max_depth: -1, 5, 10;  learning_rate: 0.03, 0.1, 0.2;  num_leaves: 15, 31, 63 | n_estimators: 100  max_depth: 10  learning_rate: 0.03  num_leaves: 15 |
| LR | C: 0.01, 0.1, 1, 10, 100;  class_weight: None, balanced;  max_iter: 2000 | C: 0.1  class_weight: balanced;  max_iter: 2000 |
| MLP | hidden_layer_sizes: (50), (100), (50,50);  activation: relu, tanh;  alpha: 0.0001, 0.001, 0.01;  learning_rate_init: 0.001, 0.01 | hidden_layer_sizes: (100)  activation: relu  alpha: 0.0001  learning_rate_init: 0.001 |
| DT | criterion: gini, entropy;  max_depth: None, 5, 10, 20;  min_samples_split: 2, 5, 10;  min_samples_leaf: 1, 5, 10;  class_weight: None, balanced | criterion: gini; max_depth: 20; min_samples_split: 10; min_samples_leaf: 1; class_weight: balanced |
| SVM | C: 0.1, 1, 10;  gamma: scale, 0.01, 0.1;  kernel: rbf;  class_weight: None, balanced | C: 0.1; gamma: scale; kernel: rbf; class_weight: balanced; probability: True |
| KNN | n_neighbors: 3, 5, 7, 11, 15;  weights: uniform, distance;  p: 1, 2 | n_neighbors:11  weights:distance;  p: 1 |

Hyperparameter optimization was performed using five-fold stratified cross-validation within the training dataset, with macro-averaged one-vs-rest AUC as the optimization metric. Mean imputation was incorporated into all pipelines. Z-score standardization was additionally applied to LR, MLP, SVM, and KNN. RF, random forest; XGB, extreme gradient boosting; GB, gradient boosting; LGBM, light gradient boosting machine; LR, logistic regression; MLP, multilayer perceptron; DT, decision tree; SVM, support vector machine; KNN, k-nearest neighbors. The final RF settings were re-audited directly against the deposited serialized model; the corrected values shown here supersede the parameter values in the previous supplementary version. The serialized RF model stored random_state=None; the public rerun script uses random_state=42 to permit deterministic refitting, while locked external predictions are generated from the deposited fitted model.

**Table S11. Performance of the locked APL-prioritized alert threshold in the development and external validation cohorts.**

| **Cohort** | **APL threshold** | **Sensitivity, % (95% CI)** | **Specificity, % (95% CI)** | **PPV, % (95% CI)** | **NPV, % (95% CI)** | **Missed APL, n** |
| --- | --- | --- | --- | --- | --- | --- |
| Development-center cohort | 0.0966 | 99.02 (98.49–99.37) | 66.23 (64.91–67.52) | 54.34 (52.73–55.93) | 99.40 (99.08–99.61) | 20 |
| Guangdong | 0.0966 | 97.66 (95.75–98.73) | 51.03 (46.35–55.70) | 66.24 (62.47–69.83) | 95.69 (92.25–97.64) | 10 |
| Wanbei | 0.0966 | 97.94 (92.79–99.43) | 48.96 (43.67–54.28) | 35.58 (30.08–41.49) | 98.80 (95.74–99.67) | 2 |
| Tongling | 0.0966 | 96.32 (94.73–97.44) | 47.91 (41.94–53.93) | 84.25 (81.68–86.52) | 81.82 (74.98–87.11) | 28 |

The threshold was selected in the development-center cohort as the highest APL probability cutoff achieving at least 99% sensitivity and was then applied unchanged to all external cohorts. External cohorts were not used for threshold selection. Confidence intervals were calculated using the Wilson method. APL, acute promyelocytic leukemia; PPV, positive predictive value; NPV, negative predictive value.

**Table S12. Variance inflation factors for the 18 predictors retained in the primary random forest model.**

| **Predictor** | **VIF** |
| --- | --- |
| Basophil percentage | 1.99 |
| Eosinophil count | 4.44 |
| Basophil count | 1.96 |
| Monocyte count | 2.47 |
| Eosinophil percentage | 4.89 |
| Neutrophil count | 1.42 |
| Lymphocyte count | 1.82 |
| Monocyte percentage | 2.59 |
| RBC | 1.80 |
| WBC | 1.56 |
| Lymphocyte percentage | 8.74 |
| PCT | 2.93 |
| PLT | 1.10 |
| Neutrophil percentage | 9.57 |
| PDW | 2.40 |
| MCH | 1.27 |
| RDW-SD | 2.33 |
| MCV | 1.75 |

VIFs were calculated in the development cohort after mean imputation. A VIF greater than 5 was considered indicative of potentially important collinearity. VIF, variance inflation factor; RBC, red blood cell count; WBC, white blood cell count; PCT, plateletcrit; PLT, platelet count; PDW, platelet distribution width; MCH, mean corpuscular hemoglobin; RDW-SD, red cell distribution width standard deviation; MCV, mean corpuscular volume.

**Table S13. Selected DeLong comparisons of SHAP-ranked reduced random forest models against the full 24-feature model using development-stage cross-validated predictions.**

| **No. of features** | **AUC** | **ΔAUC vs. 24 features** | **DeLong P value** | **Interpretation** |
| --- | --- | --- | --- | --- |
| 24 | 0.9378 | Reference | — | Full model |
| 20 | 0.9356 | −0.0022 | 0.5910 | No significant difference |
| 19 | 0.9348 | −0.0030 | 0.4649 | No significant difference |
| 18 | 0.9339 | −0.0039 | 0.2756 | No significant difference; selected model |
| 17 | 0.9046 | −0.0332 | 2.07 × 10⁻¹³ | Significantly lower |
| 12 | 0.8308 | −0.1070 | 1.38 × 10⁻⁹⁰ | Significantly lower |

The reduced models were generated by backward elimination according to the consensus SHAP ranking. For each feature, mean absolute SHAP values were calculated separately within each cross-validation training partition and then averaged across the five iterations. Each reduced RF model was compared with the full 24-feature RF model using paired development-stage cross-validated predictions. The 18-feature model was retained based on parsimony and the observed AUC trajectory. Nonsignificant DeLong results were not interpreted as evidence of equivalence or noninferiority. No external-validation data were used for feature ranking, backward elimination, or selection of the final model. AUC, area under the receiver operating characteristic curve; RF, random forest; SHAP, SHapley Additive exPlanations.

**Table S14. Class-specific performance of the final 18-feature random forest model in the three external validation cohorts.**

| **Cohort** | **Class (n)** | **AUC (95% CI)** | **Sensitivity, % (95% CI)** | **Specificity, %** | **PPV, % (95% CI)** | **NPV, %** | **F1 score** |
| --- | --- | --- | --- | --- | --- | --- | --- |
| Guangdong | APL/M3 (428) | 0.890 (0.868–0.911) | 65.42 (60.80–69.77) | 90.34 | 86.96 (82.84–90.20) | 72.64 | 0.747 |
| Guangdong | AMoL/M5 (7) | 0.929 (0.873–0.977) | 71.43 (35.89–91.78) | 92.29 | 7.04 (3.05–15.45) | 99.75 | 0.128 |
| Guangdong | ALL (428) | 0.871 (0.847–0.894) | 85.51 (81.86–88.53) | 76.09 | 77.87 (73.90–81.39) | 84.22 | 0.815 |
| Wanbei | APL/M3 (97) | 0.871 (0.832–0.906) | 60.82 (50.88–69.95) | 89.32 | 62.11 (52.06–71.21) | 88.79 | 0.615 |
| Wanbei | AMoL/M5 (116) | 0.902 (0.867–0.934) | 62.07 (52.99–70.38) | 94.34 | 80.00 (70.59–86.96) | 87.21 | 0.699 |
| Wanbei | ALL (221) | 0.890 (0.858–0.917) | 87.78 (82.81–91.47) | 74.18 | 77.91 (72.36–82.62) | 85.41 | 0.826 |
| Tongling | APL/M3 (761) | 0.865 (0.839–0.891) | 60.84 (57.33–64.25) | 90.87 | 95.07 (92.77–96.67) | 44.51 | 0.742 |
| Tongling | AMoL/M5 (32) | 0.874 (0.808–0.927) | 68.75 (51.43–82.05) | 88.51 | 16.18 (10.93–23.28) | 98.87 | 0.262 |
| Tongling | ALL (231) | 0.862 (0.839–0.885) | 84.85 (79.66–88.90) | 74.15 | 48.88 (44.02–53.76) | 94.38 | 0.620 |

Each class was evaluated against the other two classes combined. AUC confidence intervals were estimated using 2,000 class-stratified bootstrap replicates; Wilson confidence intervals were used for sensitivity and PPV. AUC, area under the receiver operating characteristic curve; PPV, positive predictive value; NPV, negative predictive value; APL, acute promyelocytic leukemia; AMoL, acute monocytic leukemia; ALL, acute lymphoblastic leukemia.

**Table S15. Calibration of the final 18-feature random forest model in the development and external-validation cohorts.**

| **Cohort** | **Class (events/N)** | **Calibration intercept (95% CI)** | **Calibration slope (95% CI)** | **Class-specific Brier score (95% CI)** | **Multiclass Brier score (95% CI)** |
| --- | --- | --- | --- | --- | --- |
| Development (5-fold OOF) | APL/M3 (2043/7077) | -0.023 (-0.093 to 0.047) | 1.570 (1.491 to 1.650) | 0.081 (0.077 to 0.084) | 0.267 (0.259 to 0.274) |
|  | AMoL/M5 (1783/7077) | -0.001 (-0.066 to 0.063) | 1.619 (1.537 to 1.701) | 0.100 (0.097 to 0.104) |  |
|  | ALL (3251/7077) | 0.022 (-0.044 to 0.087) | 1.820 (1.725 to 1.916) | 0.086 (0.082 to 0.089) |  |
| Wanbei | APL/M3 (97/434) | -0.281 (-0.565 to 0.002) | 1.019 (0.803 to 1.235) | 0.114 (0.097 to 0.130) | 0.359 (0.323 to 0.395) |
|  | AMoL/M5 (116/434) | 0.152 (-0.103 to 0.407) | 1.455 (1.158 to 1.751) | 0.111 (0.098 to 0.125) |  |
|  | ALL (221/434) | 0.077 (-0.160 to 0.315) | 1.316 (1.078 to 1.554) | 0.134 (0.118 to 0.152) |  |
| Tongling | APL/M3 (761/1024) | 2.031 (1.858 to 2.204) | 1.007 (0.865 to 1.149) | 0.236 (0.223 to 0.251) | 0.452 (0.424 to 0.479) |
|  | AMoL/M5 (32/1024) | -2.335 (-2.703 to -1.968) | 1.225 (0.878 to 1.573) | 0.070 (0.063 to 0.077) |  |
|  | ALL (231/1024) | -1.076 (-1.254 to -0.898) | 1.025 (0.884 to 1.167) | 0.146 (0.133 to 0.158) |  |
| Guangdong | APL/M3 (428/863) | 0.894 (0.720 to 1.068) | 1.154 (1.000 to 1.308) | 0.160 (0.148 to 0.173) | 0.356 (0.331 to 0.382) |
|  | AMoL/M5 (7/863) | -3.428 (-4.181 to -2.676) | 1.443 (0.690 to 2.195) | 0.050 (0.044 to 0.056) |  |
|  | ALL (428/863) | 0.119 (-0.049 to 0.287) | 1.153 (1.001 to 1.305) | 0.146 (0.134 to 0.159) |  |

Calibration in the development cohort was based on pooled out-of-fold predictions from five-fold stratified cross-validation. External probabilities were generated by the locked final model without recalibration. Calibration intercept and slope have ideal values of 0 and 1, respectively. Class-specific Brier scores are binary one-vs-rest scores. The multiclass Brier score is the mean, across patients, of the sum of squared differences between the three predicted probabilities and one-hot outcomes. Brier-score confidence intervals were estimated using 2,000 class-stratified bootstrap replicates. OOF, out-of-fold; CI, confidence interval; APL, acute promyelocytic leukemia; AMoL, acute monocytic leukemia; ALL, acute lymphoblastic leukemia.

**Figure S1. P-R curves of ten models with 24 parameters.**

Class1:M3, Class2:M5,Class3:ALL.(A)XGBoost, extreme gradient boosting. (B) LGBM, light gradient boosting machine. (C) GB, gradientboosting. (D) MLP, Multilayer Perceptron. (E) KNN, K-Nearest Neighbors. (F) DT, decision tree.(G) LR, logistic regression. (H) SVM, support vector machine. (I) RF,random forest. (J) AdaBoost, adaptive boosting.

**Figure S2. Confusion matrix heatmaps of ten models with 24 parameters.**

Class0:M3, Class1:M5, Class2:ALL .(A)XGBoost, extreme gradient boosting. (B) LGBM, light gradient boosting machine. (C) GB, gradientboosting. (D) MLP, Multilayer Perceptron. (E) KNN, K-Nearest Neighbors. (F) DT, decision tree.(G) LR, logistic regression. (H) SVM, support vector machine. (I) RF,random forest. (J) AdaBoost, adaptive boosting.

**Figure S3. Development-stage cross-validated precision–recall curves of the 18-feature RF model.**

Class 1: M3; Class 2: M5; Class 3: ALL.

**Figure S4. PR curves of the RF model with 18 parameters in the external validation set.**Class 0: M3; Class 1: M5; Class 2: ALL.

(A)The Affiliated Guangdong Second Provincial General Hospital of Jinan University

(B)Tongling People's Hospital

(C)Wanbei Coal Electric Group General Hospital

**Figure S5.**The DCA of the final RF model (18 parameters) in external validation set.

(A)The Affiliated Guangdong Second Provincial General Hospital of Jinan University

(B)Tongling People's Hospital

(C)Wanbei Coal Electric Group General Hospital

**Figure S6. Calibration plots for the final 18-feature random forest model.**

Calibration curves for APL/M3, AMoL/M5, and ALL in (A) the development cohort using pooled five-fold out-of-fold predictions and in the independent external-validation cohorts from (B) Wanbei, (C) Tongling, and (D) Guangdong. Points show observed proportions versus mean predicted probabilities in 10 quantile-based groups. The dashed diagonal indicates ideal calibration. External probabilities were generated by the locked model without center-specific recalibration. The Guangdong AMoL/M5 curve is based on only seven reference-standard cases and should be interpreted cautiously.
